# The Endoparasitoid, *Cotesia vestalis*, Regulates Host Physiology by Reprogramming the Neuropeptide Transcriptional Network

**DOI:** 10.1038/srep08173

**Published:** 2015-02-02

**Authors:** Min Shi, Shuai Dong, Ming-tian Li, Yan-yan Yang, David Stanley, Xue-xin Chen

**Affiliations:** 1 Ministry of Agriculture Key Lab of Agricultural Entomology, Institute of Insect Sciences, Zhejiang University, 866 Yuhangtang Road, Hangzhou, 310058, China; 2 Biological Control of Insects Research Laboratory, Agricultural Research Service, U.S. Department of Agriculture, 1503 S. Providence Road, Columbia MO 65203, USA

## Abstract

Endoparasitoids develop inside another insect by regulating host immunity and development via maternal factors injected into hosts during oviposition. Prior results have provided insights into parasitism-induced immunosuppression, including the neuropeptide accumulation in parasitized insects. Nonetheless, our understanding of neuropeptide influence on host development and behavior is not yet complete. We posed the hypothesis that parasitization alters expression of genes encoding pro-neuropeptides and used larvae of *Plutella xylostella* and its endoparasitoid, *Cotesia vestalis* to test our hypothesis. We prepared transcriptomes from the larval *P. xylostella* brain-CC-CA complex and identified transcripts encoding 19 neuropeptides. All corresponding cDNAs were confirmed by RACE. Our results demonstrate that parasitism significantly down-regulated, or delayed, expression of genes encoding pro-neuropeptides within 48 h post-parasitization. Changing expression of these genes may account for the previously reported decreased feeding behavior, reduced growth rates and aborted development in the host larvae. In effect, parasitization may operate at the molecular level within the CNS to create global changes in larval host biology. The significance of our finding is that, in addition to the known effects on immunity, parasitoids influence host pro-neuropeptide gene transcription. This finding reveals a new mechanism operating in host-parasitoid relationships to the advantage of the parasitoid.

Parasitoid wasps are insects that live inside or on another insect during the larval stage of their life cycle, during which they consume and eventually kill their hosts. They have evolved mechanisms to manipulate host physiology and biochemistry to create an environment that favors development of the parasitoid at the cost of the host^1^. These mechanisms involve maternal factors, including venoms, ovarian proteins, and polydnaviruses (PDVs) that are injected into the host along with eggs during oviposition. In many braconid wasps, teratocytes, cells derived from the serosal membrane of parasitoid embryos, also act in host regulation^2^. Parasitization produces a stereotypical parasitization-associated host syndrome, with symptoms including immunosuppression, decreased appetites, retarded growth rates, diverted nutritional metabolism, delayed ecdysis and abnormal metamorphosis. Insects express robust immune reactions to parasitoid eggs and research has focused on the actions of maternal factors that mediate host immunosuppression following deposition of parasitoid eggs^3^. Reports on endocrine mechanisms responsible for changing feeding behaviors and development are relatively scant^4^,^5^,^6^,^7^,^8^.

Some of the fundamental biological processes in animals, such as development, reproduction and particularly, behavior, are regulated by neuropeptides^9^. In general, neuropeptides are small peptides, from three to 70 amino acids in length. Many, but not all, neuropeptides act thorough G protein-coupled receptors (GPCRs) in the peripheral nervous system and the central nervous system (CNS). These peptides are produced in endocrine cells or neurons as precursors (pro-neuropeptides); they are converted into biologically active forms by post-translational modifications. Secreted neuropeptides have autocrine, paracrine and hormonal effects, depending on the locations and mechanisms of their specific receptors^10^. Contemporary biotechnology tools have accelerated the rate of discovery^11^ and now many insect neuropeptides and their specific roles in behavior, development and metabolism are known.

The polyphagous diamondback moth (DBM), *Plutella xylostella* (L.), is a destructive insect pest of cruciferous crops, with multiple resistances to almost every insecticide^12^. The solitary larval endoparasitoid, *Cotesia vestalis* (Haliday) (Hymenoptera: Braconidae), is widely deployed as a DBM biological control agent. It expresses the maternal immunosuppressive factors just mentioned^8^. Parasitization by *C. vestalis* suppresses host immune defenses and slows host development, promoting the success of parasitoid progeny. Although the visible influences of parasitoids in host biology are recognized, understanding the molecular mechanisms of these influences is one of the crucial needs to improve the efficacy of parasitoids in biological control programs.

To address this need, we posed the hypothesis that parasitization changes expression of DBM CNS genes encoding pro-neuropeptides. We first created a transcriptome of the larval DBM brain-corpora allata (CA)-corpora cardiaca (CC) complex (B-CA-CC), then identified all neuropeptide transcripts present in the transcriptome. We tested our hypothesis by determining the influence of parasitization on expression of genes encoding pro-neuropeptides. Our results generate a comprehensive, albeit still incomplete, catalog of the neuropeptidome of *P. xylostella* CNS and provide insights into the neuroendocrine processes affected by parasitization.

## Results

### Illumina sequencing and transcriptome reads assembly

After filtering the adaptors and low quality sequences, about 7 million 180 bp reads were obtained. These reads were subsequently assembled using SOAPdenovo software, resulting in 89,530 scaffolds (Table 1). After gap-filling and clustering, 42,441 unigenes (Table 1) were generated from these scaffolds with a mean size of 394 bp.

### Overview of B-CC-CA transcripts

**Figure 1:**
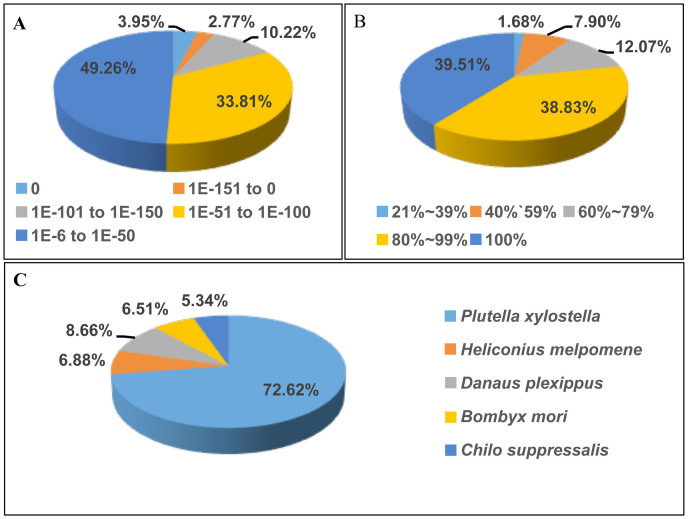
Summary of the homology search of Illumina sequences against the five lepodopteran whole genome sequences. (A) E-value distribution of BLAST hits for each unique sequence with a cut-off E-value of 1.0E^−5^. (B) Similarity distribution of the top BLASTx hits for each sequence. (C) Species distribution is shown as a percentage of the total homologous sequences with an E-value of at least 1.0E^−5^. We used the first hit of each sequence for analysis.

For functional annotation, the 42,441 unigenes were searched using BLASTx against the nr NCBI nucleotide database; 19,144 unigenes returned significant BLAST matches (E-value < 1.0E^−5^) (see Supplementary Table S3 online). The E-value distribution of the best matches against the lepidopteran genomes available in GenBank WGS database showed that 50.7% of the sequences have strong homology (E-value < 1.0E^−50^) (Figure 1A). The similarity distribution demonstrated that 90.4% of the unique sequences with best matches have a similarity, higher than 60% (Figure 1B). For the species distribution in the Lepidoptera, the highest percentage of the unigenes are matched to genes from the *P. xylostella* (72.6%), followed by *Danaus plexippus* (8.7%), *Heliconius melpomene* (6.9%), *Bombyx mori* (6.5%), and *Chilo suppresslis* (5.8%) (Figure 1C).

### Unigenes annotations

#### (a) Pro-neuropeptide genes

Nineteen neuropeptides belonging to 17 neuropeptide families were predicted (Table 2), and all of them confirmed by gene cloning. The identified pro-neuropeptide genes are presented in Table 2. The encoded proteins act in a wide range of insect biology, including lipid transport, homeostasis and development.

#### (b) Genes encoding precursor processing enzymes

Neuropeptides generally require processing by proprotein or prohormone convertases from larger precursor polypeptides to become biologically active. Unigenes identified as genes encoding precursor processing enzymes included *subtilisin-like protein*, *furin*, *angiotensin converting enzyme*, *endothelin converting enzyme 1*, *STE24 homolog* and *neuroendocrine protein 7b2* (Table 3). Some unigenes related to genes involved in synthesis of biogenic amines include *tyrosine hydroxylase*, *DOPA decarboxylase*, *tyrosine decarboxylase*, *tyramine beta hydroxylase* and *tryptophan hydroxylase*.

#### (c) Neuro-receptor genes

Unigenes identified as neuro-receptor genes are displayed in Table 4. These include 10 subtypes of *nicotinic acetylcholine receptor.* All except two are GPCRs; the other two are ligand-gated ion channels (LGICs).

### Transcription of pro-neuropeptide genes influenced by parasitization or *C. vestalis* bracovirus (CvBV)

**Figure 2:**
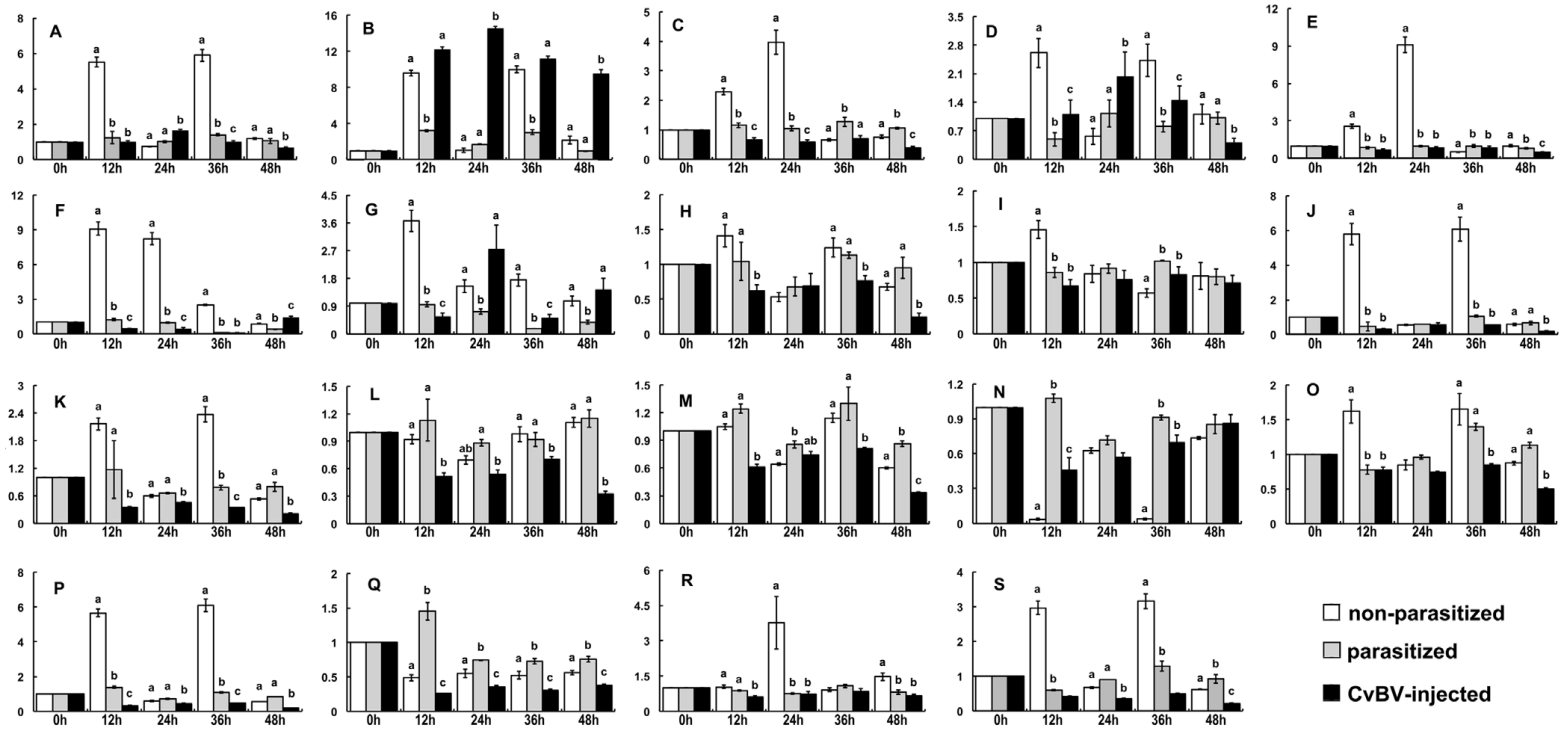
Relative abundances of pro-neuropeptide transcripts in B-CA-CC from control and experimental larvae. The relative amounts of pro-neuropeptide gene mRNAs were normalized to the abundance of *β-tubulin* mRNAs. Pro-neuropeptide transcript levels at indicated time points pp/pi were normalized to the corresponding pro-neuropeptide gene transcript at 0 hour pp/pi. (A): pro-adipokinetic hormone I gene; (B): pro-adipokinetic hormone II gene; (C): pro-A-type allatostatin gene; (D): pro- C-type allatostatin gene; (E): pro-allatotropin gene; (F): pro-bursicon subunit α gene; (G): pro-bursicon subunit β gene; (H): pro-CCHamide gene; (I): pro-crustacean cardioactive peptide gene; (J): pro-diuretic hormone gene; (K): pro-FMRFamide gene; (L): pro-ion-transport peptide gene; (M): pro-leucokinin gene; (N): pro-neuroparsin gene; (O): pro-neuropeptide F2 gene; (P): pro-short neuropeptide F gene; (Q): pro-neuropeptide-like peptide (NLP); (R): pro-prothoracicotropic hormone gene; (S): pro-tachykinin gene. X- axis: Hours post parasitization/CvBV-injection; Y- axis: Relative transcript abundance; White bar: Non-parasitized larvae; Grey bar: Parasitized larvae; Black bar: CvBV-injected larvae. Letters on the top of bars indicate the significantly different means within the relative transcript abundances at specific time points under different treatment by one-way analysis of variance (ANOVA) analysis (n = 3, P < 0.05).

Relative to non-parasitized (control) larvae, parasitization influenced the expression of genes encoding 19 pro-neuropeptides in B-CA-CCs (Fig. 2). Expression of many of these genes, for examples, pro-AKH-I, pro-BurA, pro-NPF2, pro-PTTH was down-regulated at 12 h post-parasitization (pp) or later in the time course. Expression of a couple of genes, for example the gene encoding pro-neuroparsin, was up-regulated following parasitization. Expression of two genes, pro-CCHamide and pro-ion-transport peptide, was not influenced by parasitization.

Relative to non-parasitized control larvae, injection of CvBV also influenced the expression of pro-neuropeptide in B-CA-CCs (Fig. 2). The influences of 0.1 equivalent CvBV on expression of most genes were close to or slightly stronger than the influence of natural parasitization, for examples, pro-AKH-I, pro-AT, pro-neuroparsin, pro-PTTH. There was one exception, the transcript abundance of pro-AKH II was increased after injection of CvBV.

### Parasitization influenced ecdysteroid titers but not juvenile hormone esterase (JHE) activity

**Figure 3:**
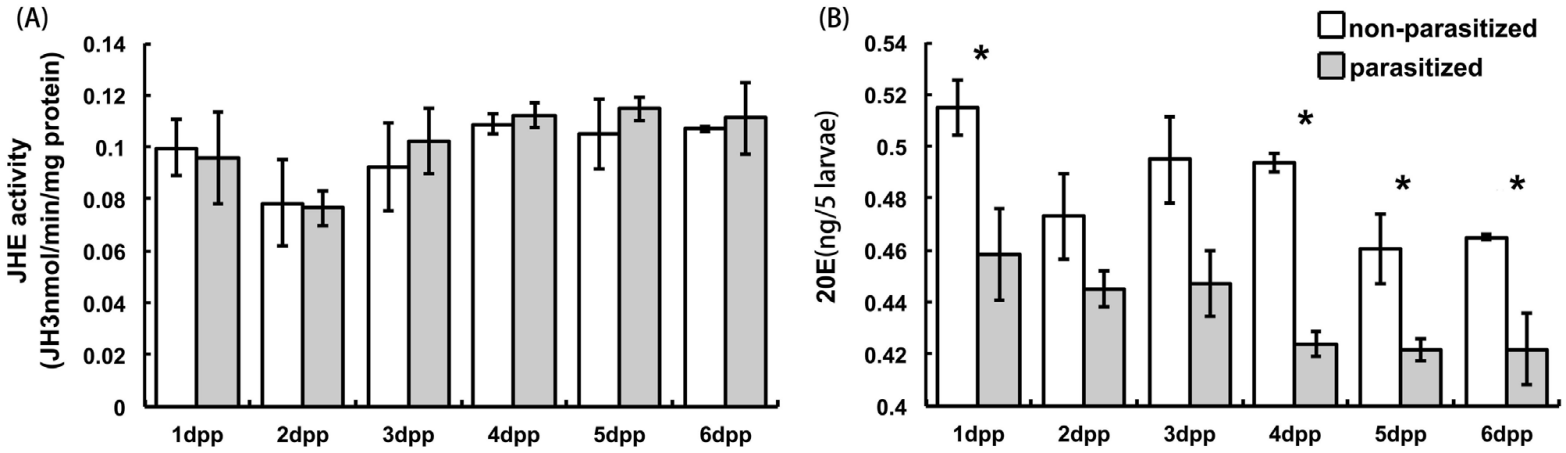
JHE activity (A) and 20E (B) titer of *P. xylostella* larvae parasitized by *C. vestalis.* White bar: Non-parasitized larvae; Grey colume: Parasitized larvae. Histogram bars annotated with an asterisk indicate significant differences by one-way analysis of variance (ANOVA) (*: P < 0.05).

JHE activity and ecdysteroid titers of non-parasitized and parasitized larvae are shown in Figure 3A and B. Parasitization did not influence JHE activity, but led to significantly decreased 20E titers.

## Discussion

The data reported in this paper strongly bolster our hypothesis that parasitization alters expression of DBM genes encoding pro-neuropeptides. The data support a straightforward argument. First, we developed a comprehensive transcriptome of the DBM B-CA-CC and used it to identify 19 genes encoding 17 neuropeptides that act in fundamental aspects of insect biology, including development, lipid transport, metabolism, regulation of feeding behavior and gut motility, and homeostasis. Second, parasitization exerted profound effects on the expression of these genes. Third, with respect to maternal factors, injected CvBV also influenced gene expression in a manner similar to parsitization. Finally, concentrations of the molting hormone, 20E, were reduced in parasitized larvae. Together, these points form a powerful statement on the influence of parasitoids on their host physiology. In particular, by influencing the expression of genes encoding pro-neuropeptides, parasitoids influence their hosts in ways that go beyond the commonly registered immunosuppression. The significance of our data is that parasitization alters the physiology of hosts at two levels: One, the suppression of immediate immune reactions to the presence of parasitoid eggs (possibly by disabling hemocytes), and two, altering host behaviors, development and on-going homeostatic physiology (by altering expression of pro-neurohormone genes). In other words, the significance of our finding is that, in addition to the known effects on immunity, parasitoids influence host pro-neuropeptide gene transcription. If our view is supported in future work, then operating at the level of CNS gene expression may be a general mechanism in metazoan host/parasite relationships.

The idea that parasitization can influence neurohormone titres has been considered in the past. Zitnan *et al*. reported that multiple neuropeptides, including PTTH, bombyxin, AT, AST, DH, EH, proctolin, and FMRFamide-like peptide, accumulated in neurosecretory cells as well as their axon terminals in the CC-CA complex after host insects, *Manduca sexta* larvae, were parasitized by the related parasitoid, *C. congregata*^1^,^13^,^14^. They suggested the accumulation of neuropeptides was related to developmental arrest of parasitized larvae. Our results might imply that the neuropeptides are produced in lower amounts, as suggested by lower mRNA expression affected by parasitization, but still accumulate in the neurosecretory cells simply because they are not secreted.

We report on substantial changes in expression of most of the neuropeptide-encoding genes in our analysis, although these genes do not form a comprehensive set of the known insect neuropeptides. Some genes not included in our findings include B-type allotostatin, ecdysis triggering hormone, myosuppressin, pheromone biosynthesis activating neuropeptide and diapause hormone^15^,^16^. To be sure, some of these are expressed in other tissues, which lies beyond the scope of our report. However, in our interrogation of the *P. xylostella* genome database (http://iae.fafu.edu.cn/DBM/index.php)^17^, we found six of the pro-neuropeptide genes in our B-CA-CC transcriptome, including AKH, AT, CCHaimde, CCAP, neuroparsin and PTTH that are not yet included in the genome database. Similarly, we found two genes (CCHamine and CCAP) not included in another *P. xylostella* transcriptome database, KONAGAbase (http://dbm.dna.affrc.go.jp/px/#tab-panel-home)^18^. Because our transcriptome database was prepared from a highly selective tissue set (B-CA-CC), all possible pro-neuropeptide transcripts are not expected. Our point is that appreciation of the broadest possible representation of pro-neuropeptide genes will require developing and interrogating multiple databases derived from several tissues. More to the point, understanding how – in detailed terms – manipulating expression of these genes advantages a parasitoid remains beyond current knowledge.

As is true for Lepidoptera generally, DBM larvae undergo a parasitization syndrome described earlier^12^,^19^,^20^. Parasitization-specific changes in the neuroendocrine system are responsible for most, if not all, aspects of the parasitization syndrome. Our data indicates that the phenotypic changes recorded in other studies^21^ may be due, directly or otherwise, to changes in expression of pro-neuropeptide genes. Our results show that parasitization reduces circulating titers of 20E, which declined significantly, but did not influence circulating JH titers because it did not influence JHE activity. We infer that the changes in pro-neuropeptide gene expression recorded here is one of the mechanisms responsible for the parasitization syndrome.

The influence of parasitization on 20E titers, reported in previous studies^1^, may be due to expression of a gene encoding pro-PTTH^1^, which was reduced by over four-fold at 24 h pp. Reducing the circulating PTTH titer may reduce 20E titers and hence delay pupal metamorphosis. Parasitization also impacted expression of genes encoding three neuropeptides that modulate CA function. It might be thought that JH would also act in the altered development in parasitized hosts, however, our experiments used late instar larvae in which JH titers are normally low. More to the point, 20E is the molt-triggering developmental hormone. Hence, the visible influence of parasitization on delayed molting is probably due to the reducing influence on 20E titers. Transcripts encoding the other canonical developmental neuropeptide, bursicon, were also far reduced compared to controls, which we take to underline the impact of parasitization on development. Near-silencing of bursicon sub-unit transcripts may also influence prophylactic immunity associated with molting^22^.

Although parasitization and, separately, experimental CvBV-injections, impacted expression of 19 genes, here we limit our discussion to a selected sub-set of these genes. Expression of AKHs, responsible for mobilizing and regulating hemolymph lipid transport^23^ or regulating digestion^24^, was decreased at 12 and 36 h pp, indicating to us lipid transport or food digestion physiology are negatively influenced by parasitism. We note, however, in the *Manduca sexta/C. congregata* system, parasitism did not change the lipophorin level of parasitized host larvae^25^, but it significantly decreased the digestive enzyme activity in midguts in the *P. xylostella/C. vestalis* system^12^. The decreased expression of pro-AKH genes reported here may be mainly responsible for low food digestion efficiency of parasitized lepidopteran hosts. Relative to pro-AKH I, the expression level of pro-AKH II was much lower in non-parasitized and parasitized larvae (see Supplementary Fig. S1 online). We note that the outcomes of our experimental virus injections differed from the influence of parasitization. Understanding the mechanisms of such difference lies beyond the scope of this manuscript.

NPF2 and sNPF regulate the food intake in *Drosophila* and possibly other insect species^26^. The transcriptional patterns of genes encoding pro-NPF2 and pro-sNPF in control larvae matched the feeding stage of each instar, indicating to us a developmentally-regulated increase in feeding activity. Parasitization and, separately, experimental CvBV injection experiments led to significantly decreased expression of both genes, which may relate to decreased feeding behavior. A similar result was reported for *D. melanogaster*, in which high expression of NPF in wandering-stage larval brain was associated with feeding, whereas loss of dNPF signaling in young transgenic larvae led to the premature feeding cessation in wandering stage larvae^27^. We infer that parasitism, and separately, the material factor, CvBV, influences feeding behavior via the influence on neuropeptide gene expression. We also note that expression of FMRFamide and tachykinin, neuropeptides that regulate gut muscle contraction in *Drosophila*, cockroaches and locusts^28^,^29^, was reduced following parasitization and, separately, CvBV-injection, indicating to us that feeding and processing food is restricted in parasitized larvae.

Parasitization/CvBV-injection also influenced homeostatic physiology in host larvae. After parasitism/CvBV-injection, expression of pro-DH was suppressed, of pro-leucokinin was increased and of ion-transport peptide was not changed, which suggests to us that parasitism/CvBV exerts contrary influences on gene expression in the B-CC-CA.

Expression of genes encoding the neuropeptides in our study varied according to the developmental stages in control larvae. Several genes, for example pro-AKH-I and pro-AST-C and pro-DH, were highly expressed in the middle of the third instars and early phase of the fourth instars. Other genes, including pro-PTTH, were expressed toward the end of the larval instars. In general, parasitization virtually silenced expression of several genes such as pro-AKH-I. On the other hand, expression of the gene encoding pro-neuroparsin was substantially increased at 12- and 36 h pp. We infer that parasitism, along with attending maternal factors did not influence all genes in the same manner.

Our data are limited to the influence of a parasitoid on expression of gene transcripts. We do not yet know the kinetics of translating these sequences into proteins, nor details of the protein actions. For a single example, AKH-I is a lipid-mobilizing factor, often associated with flight energetics. Certainly, expression in larvae raises questions – and likely hypotheses - about roles of AKH-I in larvae and about how parasitoids would be advantaged by suppressing expression of this gene. We suggest that one of the effects of parasitoid eggs and the maternal factors that accompany the eggs into host larvae takes place via influencing expression of genes encoding pro-neuropeptides. The physiological significance of altering expression of these genes will emerge from research designed to investigate separate genes.

## Methods

### Insects and parasitization

DBM pupae and parasitized larvae were initially collected from cabbage (*Brassica* spp.) fields in the suburbs of Hangzhou, Zhejiang Province, China. DBM and *C. vestalis* colonies were raised on cabbage grown at 25°C, 60–65% RH, and a photoperiod of 14:10 (L:D) h. Adult wasps were fed with 20% (vol:vol) honey-water solution and propagated using DBM larvae. *P. xylostella* larvae were used in all experiments, except as noted.

### Virus collection and injection

CvBV was collected from adult female wasps at 2 days after emergence according to the method of Beck *et al*.^30^. The amount of CvBV collected from the reproductive tract of a single adult female is defined as one wasp equivalent. In this study, 0.1 wasp equivalent of CvBV was injected into third-instar larvae, 4 hours post-ecdysis, using a hand-calibrated glass needle mounted onto a NN-153 type micromanipulator (Narishige, Japan) as described^30^.

### RNA extraction

Non-parasitized third-, fourth-instar larvae and pupae were selected randomly for dissection to prepare total RNA for transcriptome sequencing. B-CA-CCs were isolated from 3000 individuals into PBS buffer (pH = 6.8) on glass slides.

Each 3rd instar larva, 4 h post ecdysis, was exposed to one female wasp until parasitization was observed. Individual parasitized larvae were collected and fed on fresh cabbage leaves.

For each biologically independent replicate, 100 larvae were collected at selected time points post parasitization/injection (pp/pi; 0, 12, 24, 36 or 48 h pp) and B-CA-CCs were immediately isolated.

Total RNA was extracted from the B-CA-CCs using the High Pure RNA Isolation Kit (Roche Diagnostics, Germany) according to the manufacturer's manual. The RNA was treated a second time with DNase (Qiagen, Germany). Quality and concentration of the isolated total RNA were estimated by electrophoresis and NanoDrop 2000 spectrometer (ThermoFisher Scientific, Waltham, MA) at 260/280 nm.

### Library preparation

A cDNA library for transcriptome sequencing made from total RNA of non-parasitized larvae and pupae was prepared by Beijing Genome Institute (BGI, China) using a SMARTer^TM^ PCR cDNA Synthesis Kit (Clontech Laboratories, Mountain View, CA) and an Advantage 2 PCR Kit (Clontech Laboratories) following the manufacturer's instructions with slight modification. For first-strand cDNA synthesis, a 3.5 μl aliquot of total RNA (about 80 ng) was mixed with 1 μl of 12 μM 3′ SMART CDs Primer II A. The mixture was incubated at 72°C for 3 min and then 42°C for 2 min in a hot-lid thermal cycler (Eppendorf, Germany). After adding 5.5 μl Master Mix (2 μl First-Strand Buffer, 0.25 μl 100 mM DTT, 1 μl 10 mM dNTP, 1 μl 12 mM SMARTer II A Oligonucleotide, 0.25 μl RNase inhibitor, and 1 μl SMARTScribe^TM^ reverse transcriptase), the reaction was incubated at 42°C for 90 min and then terminated by heating at 70°C for 10 min. The first-strand cDNA product was used for PCR amplification using the following procedures. Two μl of first-strand cDNA combined with reaction reagents (10 μl 10× Advantage 2 PCR Buffer, 2 ml 10 μM 50× dNTP, 4 μl 12 μM 5′ PCR Primer II A, 2 μl 50× Advantage 2 Polymerase and 80 μl deionized water) were amplified using the thermal cycling program: 95°C for 1 min and variable number of cycles of 95°C for 15 s, 65°C for 30 s and 68°C for 6 min. After purification of the amplified cDNA using a QIAquick PCR Purification Kit (Qiagen), the library for transcriptome sequencing was prepared using the Illumina RNA-seq library preparation kit (Illumina, San Diego, CA) following manufacturer's recommendations.

### Illumina Sequencing and Unigene Annotation

A cDNA library was sequenced at BGI. The size of the library was approximately 200 bp/fragment and both ends were sequenced. The raw reads were cleaned by removing adaptor sequences, empty reads and low quality sequences (reads with unknown sequences ‘N’), were randomly clipped into 21 bp K-mers for assembly using de Bruijn graph and SOAPdenovo software^31^. The data sets are available at the NCBI Short Read Archive with the accession number: GBAC00000000. The assembled sequences have been deposited in the Transcriptome Shotgun Assembly (TSA) database at NCBI and can be searched using the Gene-ID listed in Supplementary Table S3 online (Nr annotated results).

### RACE sequence and peptide confirmation

The first-strand cDNAs for RACE was reverse transcribed using the ReverTra Ace qPCR RT Kit (TOYOBO, Japan) in 10 μl reaction volumes including1 μg of mixed total RNA samples extracted from non-parasitized larvae and pupae on a Pros Mastercycler (Eppendorf).

Because pro-neuropeptide gene sequences generated from the transcriptome were usually fragmented or incomplete, further confirmation of the predicted pro-neuropeptide gene candidates by 3′ RACE was required. Two sets of specific primers were designed from the partial cDNA sequences obtained from the transcriptome data (see Supplementary Table S1 online). The RACE products were cloned and sequenced, and then the candidate genes were submitted to NCBI BLASTx. BLASTx results that did not match the corresponding pro-neuropeptide genes were excluded.

### qPCR

The first-strand cDNAs for qPCR was also reverse transcribed by using the ReverTra Ace qPCR RT Kit in 10 μl reaction volumes. 1 μg of total RNA was isolated from each sample at each time point of non-parasitized, parasitized and CvBV-injected host larvae as template, separately.

Pro-neuropeptide gene specific primers were designed manually using the confirmed nucleotide sequences (GenBank accession No. KJ801914, KJ783476-KJ783493) derived from DBM B-CA-CC transcriptome data. Details of the primers are listed in Supplementary Table S2 online. The β-tubulin gene (GenBank accession No. EU127912) was used as the reference gene for normalizing the transcription levels of specific pro-neuropeptide genes. All qPCR assays were performed on an Eco^TM^ Thermal Cycler (Illumina) in 10 μl reactions. Each 10 μl reaction contained 1 μl template cDNA, 5 μl Thunderbird Sybr qPCR Mix (TOYOBO) and 0.5 μM each of the corresponding forward and reverse primers. For each pro-neuropeptide gene, three biological replicates were conducted, and generated qPCR Ct values were analyzed using the 2^−ΔΔCT^ method^32^, and further tested using one-way ANOVA method (SPSS 16.0). Here, we confirmed that the C_T_ values of the reference gene did not differ among samples of non-parasitized, parasitized and CvBv-injected host larvae (ANOVA, F = 1.62, d*f* = 14, 29, p = 0.131).

### Hemolymph preparation, juvenile hormone esterase activity and ecdystreroid titer

At 1–6 day pp, larvae were individually pierced through the cuticle with a glass pin to collect the hemolymph. For all the determinations, samples were prepared in three replications with five larvae per replicate at each time interval. Hemolymph from each replicate was collected on an ice plate and put into a glass tube with a small amount of Ethylenediaminetetraacetic Acid (EDTA) (Sigma-Alderich, Product# E6758). The hemolymph samples were stored at −70°C prior to use.

Ecdysteroid (20-hydroxyecdysteroid; 20E) in hemolymph was extracted according to Gharib and Reggi^33^ and Tawfik et al.^34^. In brief, 5 μl hemolymph was diluted into 500 μl chilled 90% methanol and partitioned against 200 μl hexane three times. The 20E partitioned into the methanol-water fraction. Quantities of 20E were determined following the method of Munyiri and Ishikawa^35^. The methanol-water fraction was evaporated to dryness using a vacuum centrifuge and dissolved in 100 μl borate buffer. Each sample was incubated with 50 μl *α*-[23, 24-^3^H(N)]- ecdysone (NEN Life Science Products, Boston, MA) 50 μl of 1:1000 polyclonal 20E antiserum and 50 μl of scintillation cocktail (Sigma-Alderich, Product# 03999) at 4°C overnight on an orbital plate shaker. Radioactivity was determined on a LS6500 liquid scintillation counter (Beckman, Fullerton, CA) with 39% counting efficiency for tritium and a standard competition curve was generated using 20E. The 20E titer is expressed as ng of 20E equivalent per larvae.

Juvenile hormone esterase (JHE) activities in hemolymph samples were analyzed using the phase partition assay according to Hammock and Spark^36^. Hemolymph samples were diluted 1:100 in 0.1 M phosphate buffer (pH7.5) with 0.1% phenylthiourea and kept on ice. A 100 μl aliquot of diluted hemolymph was incubated in 50 μl of methanol, water, and NH_4_OH (10:9:1) at 30°C for 30 min with 0.5 μM ^3^H-JH III (final concentration) (NEN Life Science Products). To stop the reaction and remove un-metabolized JH III, 250 μl of isooctane was added to the samples. The samples were thoroughly mixed and centrifuged at 1500 *g* for 10 min. The amount of radioactivity in 50 μl aliquots of the aqueous phase was estimated by scintillation counting on the liquid scintillation counter. Total protein was measured by the Bradford assay^37^.

## Author Contributions

M.S. and X.X.C. conceived and designed the experiments: M.S., S.D., M.T.L. and Y.Y.Y. performed the experiments and analyzed the data: M.S., D.S. and X.X.C. wrote and revised the manuscript. All authors reviewed the manuscript.

### Additional information

**Accession codes:** Transcript sequences from this study can be accessed through NCBI TSA database accession number GBAC00000000. cDNA sequences: GenBank accessions KJ801914, KJ783476-KJ783493.

## Supplementary Material

Supplementary Information Supplementary figures and tables

## Figures and Tables

**Table 1:** Summary of the *P. xylostella* Brain-CA-CC transcriptome

Total number of reads	7,115,762
Total base pairs (bp)	1,280,837,160
Average read length (bp)	180
Total number of contigs	429,896
Total number of scaffolds	89,530
Mean length of scaffolds (bp)	257
Total number of unigenes	42,441
Mean length of unigenes (bp)	394

**Table 2:** Overview of predicted *P. xylostella* pro-neuropeptide genes

Neuropeptides	Number of identified unigenes	Best match			
		Unigene ID	E-value	Species	Access No
Adipokinetic hormone I (AKH I)	1	16753	3E-11	*Manduca sexta*	P67788.1
Adipokinetic hormone II (AKH II)	1	2603	3E-10	*Bombyx mori*	BAG50370.1
Allatostatin A (AST-A)	1	41578	2.00E-58	*Bombyx mori*	NP_001037036.1
Allatostatin C (AST-C)	1	41728	5.00E-25	*Mythimna unipuncta*	AAA93257.1
Allatotropin (AT)	2	36309	4.00E-33	*Manduca sexta*	AAB08757.1
α-Bursicon (Bur-α)	1	39592	2.00E-58	*Manduca sexta*	Q4FCM6.1
β-Bursicon (Bur-β)	1	38012	6.00E-53	*Bombyx mori*	ABB92831.1
CCHamide	1	21065	4.00E-42	*Bombyx mori*	BAG55002.1
Crustacean cardioactive peptide (CCAP)	1	40621	3.00E-47	*Manduca sexta*	AAL39064.1
Diuretic hormone (DH)	1	9228	1E-20	*Bombyx mori*	BAG50375.1
FMRFamide	2	32972	8.00E-18	*Bombyx mori*	Q1MX22.1
Ion-transport peptide/CHH-like protein (ITP)	2	24118	4.00E-16	*Manduca sexta*	AAY29658.1
Leucokinin	1	40417	9.00E-62	*Bombyx mori*	BAG50367.1
Neuroparsin	1	30784	3.00E-25	*Bombyx mori*	BAG50366.1
Neuropeptide F2 (NPF2)	1	36645	5.00E-28	*Bombyx mori*	BAG50365.1
Short Neuropeptide F (sNPF)	1	5770	1.00E-39	*Bombyx mori*	BAG68397.1
Neuropeptide-like peptide 1 (NPL-1)	1	29354	3.00E-22	*Bombyx mori*	BAG49563.1
Prothoracicotropic hormone (PTTHH	1	10647	1.00E-44	*Spodoptera exigua*	AAT64423.1
Tachykinin	2	40209	4.00E-27	*Bombyx mori*	BAG50368.1

**Table 3:** Overview of the newly predicted *P. xylostella* genes encoding neurotransmitter precursor processing enzymes

Processing enzyme	Number of identified unigenes	Best match			
		Unigene ID	E-value	Species	Access No.
Tyrosine hydroxylase	2	42367	0	*Papilio xuthus*	BAE43824.1
Dopa decarboxylase	3	9709	1.00E-174	*Mythimna separata*	BAB68549.1
Tyrosine decarboxylase	4	17609	1.00E-126	*Drosophila melanogaster*	NP_724489.1
Tyramine Beta Hydroxylase	3	3176	1.00E-123	*Tribolium castaneum*	XP_974169.1
Tryptophan hydroxylase	3	21073	5.00E-80	*Apis mellifera*	XP_394674.2
Subtilisin-like protein	8	20138	8.00E-11	*Toxoplasma gondii*	AAK94670.1
Furin	8	20887	1.00E-167	*Trichoplusia ni*	AAT37510.1
Angiotensin converting enzyme	15	13343	3.00E-86	*Spodoptera littoralis*	ABW34729.1
Endothelin converting enzyme 1	1	32370	1.00E-12	*Nasonia vitripennis*	XP_001602211.1
STE24 homolog	2	42042	5.00E-75	*Aedes aegypti*	EAT39384.1
Neuroendocrine protein 7b2	2	19490	2.00E-35	*Dermacentor variabilis*	ACJ12615.1

**Table 4:** Overview of the newly predicted *P. xylostella* neurotransmitter receptor genes

Receptors	Classes	Subtype	Number of identified unigenes	Best match			
				Unigene ID	E-value	Species	Access No.
nicotinic Acetylcholine	LGICs	Alpha 1	1	6216	1E-104	*Bombyx mori*	ABV72683.1
		Alpha 2	3	5239	0	*Tribolium castaneum*	NP_001103423.1
		Alpha 3	5	33823	7.00E-54	*Bombyx mori*	ABV72685.1
		Alpha 4	3	18858	6.00E-92	*Bombyx mori*	ABV45515.1
		Alpha 5	4	20423	5.00E-69	*Bombyx mori*	ABV45516.1
		Alpha 6	5	16648	1.00E-89	*Plutella xylostella*	ADB84598.1
		Alpha 7	2	39426	3.00E-73	*Bombyx mori*	ABV45520.2
		Alpha 8	3	5128	9.00E-60	*Bombyx mori*	ABV72690.1
		Alpha 9	3	18150	6.00E-10	*Strongylocentrotus purpuratus*	XP_001191930.1
		Beta 1	3	27016	3.00E-36	*Bombyx mori*	ABV72692.1
muscarinic Acetylcholine	GPCRs	M1	1	17980	4.00E-22	*Danio rerio*	XP_001919160.1
		M5	1	10964	2.00E-41	*Monodelphis domestica*	XP_001370433.1
GABA	LGICs	A-type	4	40958	1.00E-109	*Plutella xylostella*	ACN52598.1
Dopamine	GPCRs	D1	1	20407	8.00E-19	*Oncorhynchus mykiss*	ACA96732.1
		D2	1	29306	7.00E-31	*Apis mellifera*	AAX62923.1
Octopamine	GPCRs	--	5	21526	1.00E-115	*Heliothis virescens*	CAA64864.1
Histamine	GPCRs	H2	1	20407	2.00E-18	*Canis familiaris*	XP_546225.2
Tyramine	GPCRs	--	3	21526	1.00E-115	*Bombyx mori*	BAD11157.1
Serotonin	GPCRs	5-HT_1_	3	12533	4.00E-80	*Drosophila melanogaster*	NP_725849.1
		5-HT_2_	4	11366	5.00E-80	*Aedes aegypti*	EAT39873.1
Tachykinin	GPCRs	--	3	3710	3.00E-40	*Aedes aegypti*	EAT41420.1
Bombesin	GPCRs	--	1	16581	5.00E-23	*Tribolium castaneum*	XP_974772.1
Neuropeptide F	GPCRs	--	5	40176	2.00E-61	*Aedes aegypti*	EAT40343.1
Allatostatin-A	GPCRs	--	2	4998	6.00E-52	*Spodoptera littoralis*	ACJ06649.1
Bursicon	GPCRs	--	1	12500	7.00E-49	*Tribolium castaneum*	ABA40401.1
Diuretic hormone	GPCRs	--	3	15611	3.00E-72	*Manduca sexta*	AAC46469.1
Leucokinin	GPCRs	--	2	21300	3.00E-28	*Drosophila melanogaster*	AAF50775.2
Short Neuropeptide F	GPCRs	--	3	31319,33333,40176	2.00E-59	*Anopheles gambiae*	ABD96049.1
Pigment dispersing factor	GPCRs	--	1	646	1.00E-06	*Drosophila melanogaster*	AAT84083.1
diapause hormone	GPCRs	--	1	2377	2.00E-39	*Bombyx mori*	NP_001036913.1
