# Supplementary material for: The Endoparasitoid, Cotesia vestalis, Regulates Host Physiology by Reprogramming the Neuropeptide Transcriptional Network
Source: Sci Rep. 2015 Feb 2;5:8173. doi: 10.1038/srep08173 (PMC4313088; doi:10.1038/srep08173)
Supplement: Supplementary Information — Supplementary figures and tables [file srep08173-s1.pdf]

Supplementary information

The Endoparasitoid, *Cotesia vestalis*, Regulates Host Physiology by Reprogramming the Neuropeptide Transcriptional Network

Min Shi[1], Shuai Dong[1], Ming-tian Li[1], Yan-yan Yang[1], David Stanley[2], Xue-xin Chen[1,*]

1. Ministry of Agriculture Key Lab of Agricultural Entomology, Institute of Insect Sciences, Zhejiang University, Hangzhou, China
2. Biological Control of Insects Research Laboratory, Agricultural Research Service, U.S. Department of Agriculture, Missouri, USA

***Corresponding author:** Xue-xin Chen, Ministry of Agriculture Key Lab of Agricultural Entomology, Institute of Insect Sciences, Zhejiang University, 866 Yuhangtang Road, Hangzhou, 310058, China, email: xxchen@zju.edu.cn

Funding: this work was jointly by the 973 Program (2013CB127600), the National Science Fund for Innovative Research Groups (31321063), the Zhejiang Science Fund for Distinguished Young Scholars (R3110049) and the Fundamental Research Funds for the Central Universities.

Table S1. Specific primers for neuropeptide precursor-related gene for 3' RACE

Neuropeptides	Primers (5' → 3')	
	Forward 1	Forward 2
Adipokinetic hormone I	CATGGGGAAACTCTTCTAGTCAGCC	CTTCTAGTCAGCCAGTTAATTGAGC
Adipokinetic hormone II	ATGCACAAAATAAGTTTCCTCT	GCACAAAATAAGTTTCCTCTTCG
Allatostatin A	CGCTACAAAGTATGCCGATT	GGAACTTTTGAGTCGTGCTT
Allatostatin C	CAGGACGGGATGCTATCTTCA	CATTGACCTCAACCAGGACGG
Allatotropin	ACCGCGTCAGTGCGATTGTG	CGTCAGTGCGATTGTGCAAAGT
Bursicon subunit α	GATCAGTGCTTTCCTTTAAGTCT	CTTCAGTTAACCAGGTAATCATT
Bursicon subunit β	TGCTACAGCATACAGCTCTCACA	CACAATTTTTCAGAATAAGTTAGGT
CCHamide	CGCAGCTTCCTACGAACACCT	AATGAGTTATGCCGCGGGTT
Crustacean cardioactive peptide	CTGGTCTTCCGTCTGAGGATA	GCCATCTTGTTGCAAAGTGACA
Diuretic hormone	TAGGCCATTAGACTGGGGTGTA	CGCATGAAATCTGTAGAGTGGGA
FMRFamide	CATACCCCAGCATGTGGAGCC	CGCCGCAGCGCTATTGATA
Ion-transport peptide/ CHH-like protein	AATACGACTCACTATAGGGCAAGCAGTGG	AGTACATGGGGACCGCCACAATAGC
Leucokinin	ATCGCGGTGAAATCTCTTTGATGTCC	CCAGGAACATTTGGAACGGCATCGCT
Neuroparsin	TCCGAGCGCAGACCTTACAGCAT	GCCACCAGCCAGACATCTCTACATCA
Neuropeptide F2	GAAGAGTTCGCAGCAACAATGAGA	CTCCTCTCCACCATCCTGCTCA
Short Neuropeptide F	CACCAACCGCTGAATACTCATAGA	TGGCGCAGTCAGCCCAAGAG
Neuropeptide-like peptide	GAAGAGTTCGCAGCAACAATGAGA	CTCCTCTCCACCATCCTGCTCA
Prothoracicotropic hormone	TTGAGTGCCGAGGCGTCCAGAT	AGGCGTCCAGATATGAAGAAGGTA
Tachykinin	AGGGCTCAAATGAACGGATTCT	GGATTCTTCGGAATGCGTGGC

Table S2. Specific primers for neuropeptide genes for real-time qPCR

Neuropeptides	Primers (5’ → 3’)	
	Forward	Reward
Adipokinetic hormone I	ACGTTTACGTCAAGTTGGGGA	CGGTTGTCAAAGACGCGTAG
Adipokinetic hormone II	GAGTTTGTAAATCGCAATGAGAGTC	CGCCTTTTTCGTGTTTTTCTG
Allatostatin A	GAGCGAGCACATCCACGAAC	AGTCTTCCTGGTCGATGTTGTTG
Allatostatin C	TTTGCGGTAACATTCCTCTCT	GCGAATAACTGTCTGATAGAACCA
Allatotropin	GCTGATGACAAGACCGTGGAG	CTGAAGATAGCATCCCGTCCTG
Bursicon subunit α	GCAGCGGCGATGTTAGTGT	CTCCTCGTCAAGGCGAATGC
Bursicon subunit β	GTACAAGCTATGTTCAGGTTTCGG	GGACGACACATACACTCAAGCG
CCHamide	TTATACTGAACCTGACCTGTGCC	CAGCGACAAAATAGTTATCAATGG
Crustacean cardioactive peptide	CGATACCCAGAAACTACGAGCAG	GCCGAGAGTGTCTTCGTCAATG
Diuretic hormone	AGACAGACTAGCCAAAGATGTGATA	CGCACTATGTGTTAAAAACTATTGT
FMRFamide	GACAGCGAAGAGTTGAATGACAC	CATCTCACTTCAACAAGCACGA
Ion-transport peptide/ CHH-like protein	CTACAGCTTACGCTATGCAAAAAC	CTGACACTCGACTGTGAAGGC
Leucokinin	GAAGAAGGATGGCAAACGG	ACGGACAGGAGGTGGAAACT
Neuroparsin	TAGAGATGTCTGGCTGGTGGC	GGTGGTTGTTAGGGCGTGTT
Neuropeptide F2	AGAGTTCGCAGCAACAATGAG	TACTGAAGCGTCTGCTATGTGC
Short Neuropeptide F	CTTGCTAACGGATCTTACAACTACA	AGGGGTATATATTTGAATGTTGTCG
Neuropeptide-like peptide	GATGTAACCGCTCATGCTCTT	CAGAACCAGCAGAGTGACAAGA
Prothoracicotropic hormone	CACCACCAAACTCTGCTCGC	GTGACTGGCTTCTTGAGGGCTAC
Tachykinin	AATGCGTGGCAAGAAGTGG	TGAAATAATGATTCTGGCAACTACA
β-tubulin	TGGCACCACACCTTCTAC	CATGATCTGGGTCATCTTCT

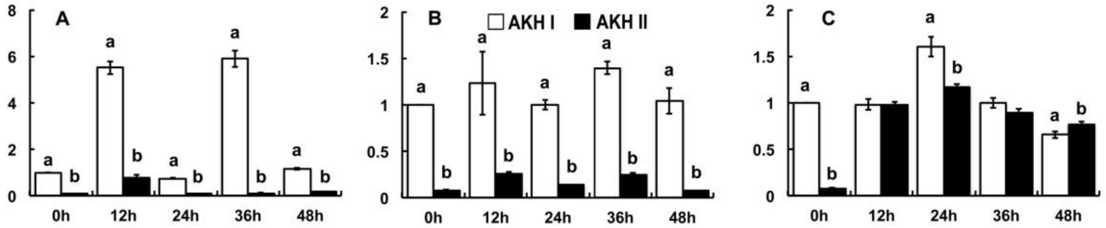

Figure S1. Comparison of the transcriptional abundances of pro-adipokinetic hormone I/II in B-CA-CC. A: non-parasitized; B: parasitzed; C: CvBV-injected. The relative amounts of pro-neuropeptide gene mRNAs were first normalized to the abundance of β-tubulin mRNAs, then these normalized value was secondly divided by the amount of the pro-AKH I at 0 h under corresponding treatment. “X” axis: Relative transcript abundance; “Y” axis: Hours post parasitization/CvBV-injection; White bar: pro-AKH I; black bar: pro-AKH II. Letters on the top of bars indicate the significantly different means within the relative transcript abundances at specific time points by one-way analysis of variance (ANOVA) analysis (n=3, $P < 0.05$).
